# Role of preoperative MR volumetry in patients with renal cell carcinoma for prediction of postoperative renal function after radical nephrectomy and nephron sparing surgery

**DOI:** 10.1590/S1677-5538.IBJU.2019.0217

**Published:** 2020-01-10

**Authors:** Hira Lal, Paritosh Singh, Priyank Yadav, Anuradha Singh, Uday P. Singh, Sanjoy K. Sureka, Rakesh Kapoor

**Affiliations:** 1 Department of Radiodiagnosis Sanjay Gandhi Post Graduate Institute of Medical Sciences Lucknow India Department of Radiodiagnosis, Sanjay Gandhi Post Graduate Institute of Medical Sciences, Lucknow, India;; 2 Department of Urology and Renal Transplantation Sanjay Gandhi Post Graduate Institute of Medical Sciences Lucknow India Department of Urology and Renal Transplantation, Sanjay Gandhi Post Graduate Institute of Medical Sciences, Lucknow, India

**Keywords:** Glomerular Filtration Rate, Magnetic Resonance Imaging, Nephrectomy

## Abstract

**Purpose:**

Preoperative computed tomography or magnetic resonance (MR) imaging are commonly used for radiological evaluation of renal cell carcinoma (RCC) before radical nephrectomy or nephron sparing surgery(NSS). This study aimed to assess the role of MRI for predicting postoperative renal function by preoperative estimation of renal parenchymal volume and correlation with glomerular filtration rate (GFR).

**Materials and Methods:**

A prospective observational study was conducted from February 2015 to October 2016 at a tertiary care hospital in northern India. MR imaging was done on 3 Tesla MR scanner (Signa Hdxt General Electrics, Milwaukee, USA). MR volumetry was used to estimate the renal parenchymal volume. GFR was measured in all patients using Tc99m Diethyl-triamine-penta-acetic acid using Russell’s algorithm. Such measurement was done preoperatively, and postoperatively 3 months after surgery.

**Results:**

30 patients with suspected RCC underwent NSS (n=10) and radical nephrectomy (n=20). Median tumour volume was 175.7cc (range: 4.8 to 631.8cc). The median volume of the residual parenchyma on the affected side was 84.25±41.97cc while that on the unaffected side was 112.25±26.35cc. There was good correlation among the unaffected kidney volume and postoperative GFR for the radical nephrectomy group (r=0.83) as well as unaffected kidney volume, total residual kidney volume and residual volume of affected kidney with postoperative GFR for the NSS group (r=0.71, r=0.73, r=0.79 respectively; P <0.05).

**Conclusion:**

Preoperative residual parenchymal volume on MR renal volumetry correlates well with postoperative GFR in patients with RCC undergoing radical nephrectomy or NSS.

## INTRODUCTION

Renal cell carcinoma (RCC) is the most common renal malignant tumor in adults (1). Its incidence has constantly increased over the last decades and currently 150.000 new cases are diagnosed every year (2). Once characterized by the classical triad of abdominal mass, flank pain and hematuria, the majority of cases are now diagnosed incidentally, these incidental masses are usually smaller compared to the historically large tumours (3). The surgical approach is directed towards removing the tumour in such a way that the maximum possible mass of the nephrons is salvaged. Consequently, nephron sparing surgery (NSS) has become the treatment of choice for small, localized RCC (4).

Preoperative radiological evaluation and staging of RCC has the following aims: (a) to evaluate the tumor for involvement of adjacent organs, lymph nodes and/or tumor thrombus, and (b) to assess the vascularity of the tumor in terms of number of arteries/veins and parasitic vessels. Using these parameters, it is possible to plan a surgical approach, however, these parameters do not help to know how much actual renal parenchyma is salvageable. Magnetic resonance imaging (MRI) is a non-invasive diagnostic tool which has the additional benefit of lack of ionizing radiation and need for iodinated contrast agent compared to computed tomography (CT), and it can be used to estimate the renal parenchymal volume (RPV) preoperatively. It is imperative that with such advantages, MRI could be a potential tool for studying the anatomy of kidneys in patients with RCC and the RPV thus calculated could quantify the nephron function in terms of glomerular filtration rate (GFR). Hence, the objective of this study was to assess the role of MRI in preoperative estimation of RPV in patients with RCC and correlate RPV with GFR to predict postoperative renal function.

## MATERIALS AND METHODS

This prospective observational study was conducted at a tertiary care hospital in northern India from February 2015 to October 2016. The study was approved by the Institution’s Ethics Committee (IEC code: 2015-26-MD-EXP) and all patients signed the consent before enrolment. Inclusion criterion was patients aged 18 years and older who were diagnosed with renal mass on ultrasonography and were planned for NSS or radical nephrectomy. Exclusion criteria were: (a) patients with claustrophobia, (b) presence of allergy to intravenous gadolinium contrast medium, (c) medical contraindications to MRI (such as orthopnoea in patients with congestive heart failure), (d) presence of MRI incompatible cardiac pacemaker, and (e) patients who were found to have tumors other than RCC on histopathological examination. The sample size was estimated assuming type one error of 0.05, a type two error of 0.02 and an expected correlation coefficient of 0.50, which yielded a value of 29 (5).

MR imaging was done on a 3 Tesla MR scanner (Signa Hdxt General Electrics, Milwaukee, USA). The coil used was phased array Torso PA (Body Coil) with patient in supine position. Sequences were entirely breath-held with field coverage of area of interest and imaging protocol included pre and post contrast sequences in axial, coronal and sagittal planes. The contrast used was Gadobenate dimeglumine (Multihance^®^) and the dose was 0.1mmol/kg.

GFR was measured in all patients by plasma clearance method. GFR estimation was done once before surgery and then 3 months after surgery. Plasma clearance of Tc99m Diethyl-triamine-penta-acetic acid (Tc99m-DTPA) was calculated using two venous blood samples at 60 and 180 minutes after injection of 1mCi Tc99m-DTPA. Russell’s algorithm was used to calculate the GFR and plasma was made protein free prior to measurement of radioactivity (6).

### MR Volumetry

**Figure 1:**
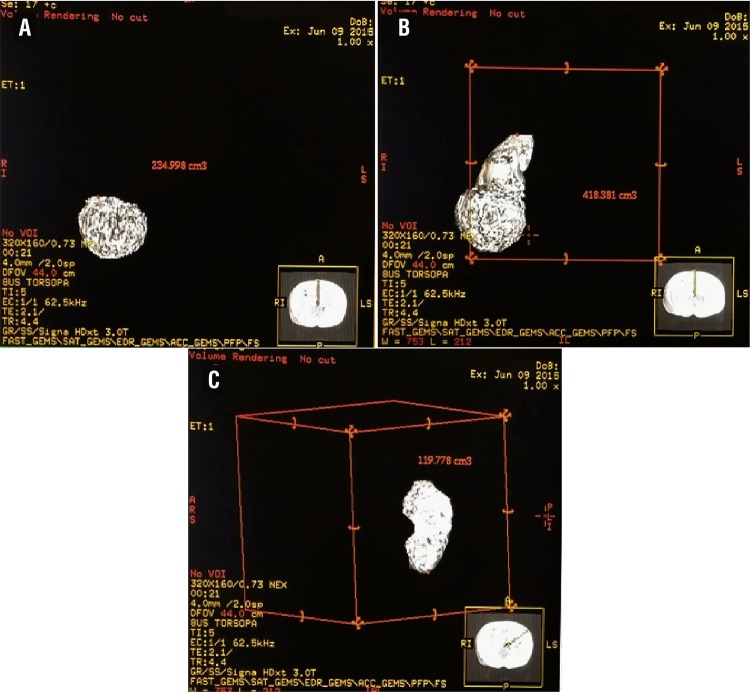
MR 3D volume reconstructed images showing (A) tumour volume, (B) affected kidney volume with tumour, and (C) unaffected kidney volume, in a patient planned for radical nephrectomy.

MRI based RPV was estimated using advanced GE workstation (Figure-1). The nonfunctional renal tissues (such as renal sinus) were excluded from these measurements. Patients were divided into two groups: one who underwent NSS and other who underwent radical nephrectomy. In NSS patients, parenchymal volume of both affected and normal kidneys correlated with postoperative GFR calculated using Tc99m-DTPA. Similarly, in radical nephrectomy patients, parenchymal volume of normal kidney correlated with postoperative GFR calculated using Tc99m-DTPA.

### Statistical analysis

MRI based preoperative RPV correlated with postoperative renal function (i.e. GFR) using Pearson’s correlation coefficient (0.0-0.2, poor; 0.2-0.4, fair, 0.4-0.6, moderate, 0.6-0.8, good, 0.8-1.0, excellent) using SPSS. A p-value of 0.05 or less was considered as statistically significant.

## RESULTS

During the study period, 30 patients with suspected RCC underwent MR volumetry and GFR estimation as per protocol described earlier. Out of these, 10 patients underwent NSS while 20 patients underwent radical nephrectomy. The mean age at diagnosis was 51.56±14.74 years. There were 22 males and 8 females.

**Figure 2:**
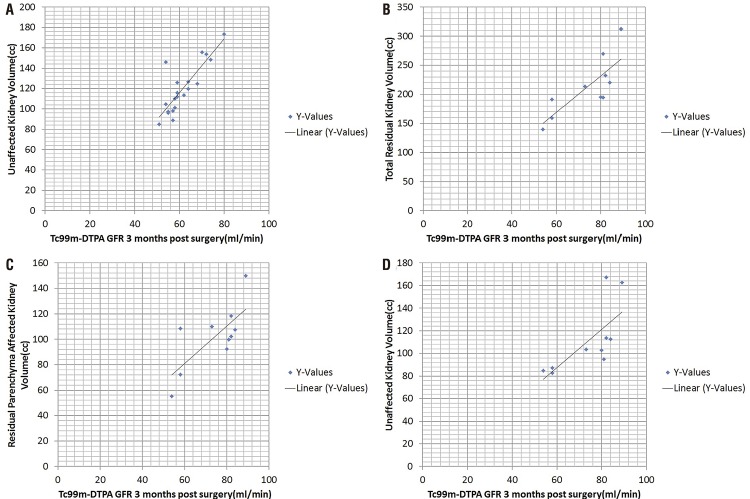
Correlation analysis of (A) GFR and unaffected kidney volume on imaging 3 months after radical nephrectomy surgery, (B) GFR and total residual kidney volume (affected+unaffected kidney) on imaging 3 months after Nephron sparing surgery, (C) GFR and residual parenchyma affected kidney volume on imaging 3 months after Nephron sparing surgery, and (D) GFR and unaffected kidney volume on imaging 3 months after nephron sparing surgery.

The median tumour volume in these patients was 175.70±178.72cc (range: 4.80 to 631.80cc) while the median residual parenchyma of the affected kidney was 84.25±41.97cc (range: 23.20 to 183.40cc). The median residual parenchyma of the unaffected kidney was 112.25±26.35cc (range: 82.8 to 173.4cc). The mean serum creatinine was 1.2±0.4mg/dL before surgery and 1.1±0.3mg/dL at 3 months after surgery (P=0.721). The mean preoperative GFR as estimated using Tc99m-DTPA was 74.60±17.18mL/min/1.73m^2^ while the mean GFR at 3 months after surgery was 65.70±11.19mL/min/1.73m^2^ (P=0.253). The correlation of the GFR with kidney volume is presented in Figure-2. Table-1 shows the correlation between the estimated radiological volume and Tc99m-DTPA GFR (P<0.05).

**Table 1:** Correlation between estimated radiological volume on MRI and Tc99m-DTPA Glomerular filtration rate (GFR).

Surgical Procedure	Estimated Radiological Volume	Pearson’s Correlation Coefficient (vs. Tc99m-DTPA GFR)
Partial Nephrectomy	Total Residual Kidney Volume (Affected+Unaffected kidney)	0.79
	Affected Kidney Volume	0.73
	Unaffected Kidney Volume	0.71
Radical Nephrectomy	Unaffected Kidney Volume	0.83

## DISCUSSION

The only curative treatment for RCC is its complete surgical excision. Traditionally, radical nephrectomy has been the gold standard but recently, as more tumors are diagnosed earlier when they are still smaller, nephron sparing surgery (NSS) has become very popular. NSS is considered the standard surgical treatment of small renal tumors (7). Preoperative cross-sectional imaging with either CT or MRI is paramount in determining the feasibility of NSS. Other than detailing the tumor size, location and relationship with the collecting system, MRI also detects the pseudo-capsule, its thickness and integrity which is particularly associated with small renal tumors and serves as a good indication for partial nephrectomy (8).

The volume of solid visceral organs can be accurately measured with CT and MRI with error less than 3% (9). With newer software programs, nonfunctional renal tissues such as renal sinus and tumor can be excluded from these measurements, thus providing a more accurate determination of functional parenchyma. This software technology is available with most MRI imaging systems and can be learned and executed easily, as was done in the present study.

The correlation between CT renal volume measurements and renal function has been studied previously. Morrisroe et al. evaluated the utility of CT based RPV measurements as a surrogate marker for differential renal function (10). In their study, CT determined renal volume measurement strongly correlated with differential renal function measured on nuclear renal scans in normal and chronically obstructed kidneys. These volume measurements were calculated in non-contrast as well as contrast images. Ng et al. demonstrated a correlation between differential renal volume calculated on non-contrast CT imaging and differential creatinine clearance measured in 24-hour urine sample in obstructed kidney (11). Johnson et al. found that in potential transplant donors, RPV determined on contrast enhanced CT scans, correlated strongly with GFR (r=0.62) (12). On MRI, Di et al. demonstrated measurement of renal volume using respiratory-gated MRI in subjects without known kidney disease and concluded that renal volume measurement by MR imaging is highly reproducible (13). Coulam et al. studied measurement of renal volumes with contrast-enhanced MRI and an excellent correlation was found between MRI measurement of total renal parenchymal volume and autopsy volume (R^2^=0.86) and weight (R^2^=0.90) (14).

The utility of CT and MRI based RPV measurements in predicting postoperative renal function following radical nephrectomy and NSS has been scantily studied. There is an increasing body of evidence suggesting that preserved RPV and other non-modifiable factors are the primary determinants of long term postoperative renal function (15). Sorberllini et al. found that the percentage change in renal volume was associated with postoperative renal insufficiency (16). When we correlated the preoperative residual renal volume with postoperative Tc99m-DTPA GFR three months after surgery, we found an excellent correlation among volume of the unaffected kidney and postoperative GFR (r=0.83) for the radical nephrectomy group. For the partial nephrectomy group, we correlated the volume of unaffected kidney, the residual volume of the affected kidney and total volume of unaffected kidney plus residual volume of affected kidney separately with the postoperative GFR and found good correlation (r=0.71, r=0.73, r=0.79 respectively, P <0.05). Kuru et al. evaluated role of volumetry in prediction of early renal function after nephron sparing surgery in 35 patients with a solitary kidney (17). Tumor volumetry was performed on CT or MRI with the Medical Imaging Interaction Toolkit. Follow-up data included renal function for 3 years. Mean tumor volume on imaging was 27.5±48.6cc which was substantially lower than the tumor volume in the present study. They found a correlation between renal function estimated by Modification of Diet in Renal Disease (MDRD) equation and residual kidney volume on imaging 1-week post-surgery while mid- and long-term renal function did not have such correlation. They concluded that renal volumetry may predict early renal function after NSS. Jeon et al. performed CT based volumetry for patients with radical and partial nephrectomy and correlated the residual renal volume with GFR estimated using modified Cockcroft-Gault formula. They found that residual renal volume and GFR correlated, although not as strongly as reported by Kuru et al. (r=0.53 for radical nephrectomy and r=0.42 for partial nephrectomy) (18). In the present study, we have correlated the RPV with Tc99m-DTPA based GFR which is more accurate than MDRD equation or modified Cockcroft-Gault formula. Liu et al. too correlated the RPV determined via CT with Tc99m-DTPA based GFR and found that the two correlated well (5). Kunzel et al. performed CT based volumetry in patients with renal mass who were planned for nephrectomy (19). In these patients, preoperative CT based RPV measurements were found to be independently associated with the development of CKD after surgery. Isotani et al. reported similar results in their cohort of 60 patients undergoing radical nephrectomy who underwent renal volumetry using CT and GFR measurement by MDRD equation (r=0.54) (20). Besides Kuru et al., only three more studies have correlated MRI renal volumetry with renal function/GFR for patients undergoing nephrectomy (Table-2) (21-23). However, in one of these studies, patients other than those with renal mass were also included. Further, in these studies, it is unclear as to how many patients in these studies actually had volumetry done on MRI and not CT. Nevertheless, a significant correlation was reported between the predicted and actual function renal volume and renal function after surgery. In contrast, the present study focuses on patients with renal mass only, CT volumetry has not been done because the objective of the study was to assess the role of MR volumetry and the method used for GFR estimation is most accurate unlike previous studies. Hence, although the sample size in the present study may appear small, it includes only MR volumetry.

**Table 2:** Previous studies correlating MRI based renal volumetry with renal function.

Authors	Year	No. of patients	Imaging modality	GFR measurement method	Correlation coefficient (r)
Tanaka N (21)	2004	102	CT, MRI#	Cockroft-Gault formula	0.86 (RN), 0.98 (NSS)
Kuru TH (17)	2014	35	CT, MRI#	MDRD equation & CKD-EPI formula	0.75
Hosokawa Y (22)	2014	83*	CT, MRI#	MDRD equation	0.46
Mibu H (23)	2015	139	CT, MRI#	MDRD equation	0.79 (RN), 0.79 (NSS)
Present study	2019	30	Only MRI	Tc 99m-DTPA renography	0.83 (RN), 0.79 (NSS)

**RN =** Radical Nephrectomy; **NSS =** Nephron Sparing Surgery; **MDRD =** Modification of Diet in Renal Disease Study; **CKD-EPI =** Chronic Kidney Disease Epidemiology Collaboration; **DTPA =** Diethyl-triamine-penta-acetic acid

*also includes patients other than those with renal mass

#all patients did not undergo MRI

The present study is limited by the small number of patients. The decision of radical nephrectomy versus NSS was primarily guided by the appearance of the tumor on MRI and its relation with the collecting system, main vessels, etc. rather than the volumetry data. The ideal application of the outcomes of such a study would be as a tool to guide the decision of NSS in a patient with borderline renal function where preservation of renal parenchyma is not only desirable but essential. The strength of this study is that this is a unique study which has correlated the residual renal volume with postoperative renal function using MRI, while most of the existing literature has such correlation performed on CT which has radiation concerns, especially for NSS where frequent imaging is needed in follow-up. Secondly, while most of the studies have used creatinine based estimates for GFR, we have used Tc99m-DTPA increasing the accuracy of GFR estimation.

In conclusion, the preoperative residual renal parenchymal volume on MR renal volumetry correlates well with the postoperative renal function.These measurements will enhance the surgeon’s ability to provide the benefit of nephron preservation without compromising the surgical outcomes.
